# Evaluation of the Temple Touch Pro™ noninvasive core-temperature monitoring system in 100 adults under general anesthesia: a prospective comparison with esophageal temperature

**DOI:** 10.1007/s10877-022-00851-z

**Published:** 2022-04-04

**Authors:** Anselm Bräuer, Albulena Fazliu, Ivo F. Brandes, Falk Vollnhals, Rolf Grote, Matthias Menzel

**Affiliations:** 1 Department of Anesthesiology, University Medical Centre Göttingen, Robert-Koch-Str. 40, 37075 Göttingen, Germany; 2 Department of Anesthesiology, Emergency Medicine, Intensive Care Medicine and Pain Therapy, Klinikum Wolfsburg, Wolfsburg, Germany

**Keywords:** Body temperature, Core temperature, Adult, Anesthesia, Monitoring

## Abstract

Perioperative hypothermia is still common and has relevant complication for the patient. An effective perioperative thermal management requires essentially an accurate method to measure core temperature. So far, only one study has investigated the new Temple Touch Pro™ (Medisim Ltd., Beit-Shemesh, Israel). during anesthesia Therefore, we assessed the agreement between the Temple Touch Pro™ thermometer (TTP) and distal esophageal temperature (T_Eso_) in a second study. After approval by the local ethics committee we studied 100 adult patients undergoing surgery with general anesthesia. Before induction of anesthesia the TTP sensor unit was attached to the skin above the temporal artery. After induction of anesthesia an esophageal temperature probe was placed in the distal esophagus. Recordings started 10 min after placement of the esophageal temperature probe to allow adequate warming of the probes. Pairs of temperature values were documented in five-minute intervals until emergence of anesthesia. Accuracy of the two methods was assessed by Bland-Altman comparisons of differences with multiple measurements. Core temperatures obtained with the TTP in adults showed a mean bias of -0.04 °C with 95% limits of agreement within − 0.99 °C to + 0.91 °C compared to an esophageal temperature probe. We consider the TTP as a reasonable tool for perioperative temperature monitoring. It is not accurate enough to be used as a reference method in scientific studies, but may be a useful tool especially for conscious patients undergoing neuraxial anesthesia or regional anesthesia with sedation.

*Trial registration* This study was registered in the German Clinical Trials Register (DRKS-ID: 00024050), day of registration 12/01/2021.

## Introduction

Perioperative normothermia is an important quality metric in anesthesia [1]. Still, despite significant efforts, perioperative hypothermia is yet common [2, 3] and has relevant complications like increased blood loss [4–6], higher amount of perioperative transfusions [2, 4, 7–9] and surgical site infections [9–12].

An adequate and effective perioperative thermal management requires essentially an accurate method to measure core temperature before induction of anesthesia, during and after anesthesia. Therefore, perioperative core temperature monitoring is recommended by several guidelines [13–15]. The ideal temperature measurement method should provide reliable, reproducible values [16]. In addition, the device should be small, easy to use, comfortable, fast, continuous, noninvasive, low energy consuming, affordable [17] and should be able to measure core temperature in awake patients. In contrast to many conventional sites of accurate core temperature monitoring (blood, esophagus, nasopharynx or bladder) new temperature monitoring devices [18, 19] are totally non-invasive, thus allowing continuous monitoring of core temperature from the time when the patient enters the operating room until the patient leaves PACU. These devices also allow to monitor core temperature in awake patients under spinal anesthesia [20].

So far, only one study has investigated the new Temple Touch Pro™ (Medisim Ltd., Beit-Shemesh, Israel). during anesthesia [21]. The system uses an algorithm to estimate core temperature from the temperature measurements made from cutaneous and environmental sides of an insulator with known thermal properties [21]. Additional studies are required to evaluate if this new device is accurate enough for perioperative thermal management in adult patients undergoing surgery with general anesthesia. Therefore, we assessed the agreement between the Temple Touch Pro™ thermometer (TTP) and distal esophageal temperature (T_Eso_) in adult patients.

## Methods

Institutional Review Board approval for this prospective multi-center observational study was granted by the local ethics committees (Ethics committee of the University Medical Centre Göttingen, No. 19/11/20 and ethics committee of the Medical School of the Martin Luther University Halle Wittenberg, Germany, No 2021-055). Written informed consent was obtained from the patients before enrollment. The study was registered in the German Clinical Trials Register (DRKS-ID: 00024050) on 12th of January 2021 before enrollment of the first patient. We followed STROBE guidelines for reporting of observational studies [22]. The inclusion criteria were age > 18 years and a planned duration of anesthesia of more than 60 min. Exclusion criteria were an esophageal disease that forbids the placement of an esophageal temperature probe, cardiothoracic operations and operations in which the surgical field would have been impeded by the esophageal probe and participation in another interventional study.

## Study protocol

In total 100 patients were studied in two centers (University Medical Center Göttingen, Klinikum Wolfsburg).

Before induction of anesthesia the TTP sensor unit was attached to the skin above the temporal artery and connected to the monitor connecting unit. The temperature data of the monitor connecting unit were then transferred to the patient monitoring system.

After induction of anesthesia an esophageal temperature probe (RÜSCH Temperature Sensor™, Teleflex Medical, Athlone, Ireland) was placed in the distal esophagus. Insertion depth was calculated for each patient according to the formula of Mekjavic [23]. The temperature probe was then connected to the patient monitoring system.

Recordings started 10 min after placement of the esophageal temperature probe to allow adequate warming of the probes. Pairs of temperature values were recorded in five-minute intervals until emergence of anesthesia began. Then the esophageal temperature probe and the TTP sensor unit were removed. After removal of the TTP sensor the skin was inspected to detect possible adverse effects like burns or erythema.

The usual thermal management of the patients was not changed by the study. In general thermal management consisted of active prewarming with forced-air before induction of anesthesia, warming during anesthesia with forced-air and infusion warming when larger amounts of fluids were used. In some patients conductive warming was used.

In addition to the temperature data the following parameters were documented: age, weight, height, sex, indication for surgery, operative procedure, ASA status, anesthesia method (TIVA, balanced anesthesia, use of an epidural catheter), warming method and the occurrence of any reactions or lesions to the skin.

## Data analysis and statistical analysis

We compared the temperature data obtained by the TTP with data obtained with the esophageal temperature probe using the Bland-Altman comparison of differences with multiple measurements [24]. The sample size of 100 patients was considered to be sufficient to demonstrate a clinically meaningful difference, as no formal rules for power calculations for this method exist. Further, we calculated the proportion of all differences that were within a predefined threshold of ± 0.5 °C of T_Eso_ [21] and Lin’s concordance correlation coefficient to assess the agreement between pairs of observations.

Then we calculated sensitivity, specificity, positive and negative predictive values for the detection of hypothermia and hyperthermia for the TTP. Hypothermia was defined as T_Eso_ < 36 °C and hyperthermia was defined as T_Eso_ > 38 °C.

In addition, we performed an error grid analysis [19] to determine if measurement differences would lead to wrong clinical decisions. The Zones were defined as follows:

Zone A begins with an area of a ± 0.5 °C error on either side of a perfectly accurate measurement between T_Eso_ and the temperature measured by the TTP. Measurement errors smaller than ± 0.5 °C are considered by most authors as clinically irrelevant. In addition, if both measurements indicate hypothermia < 36 °C or hyperthermia > 38 °C the absolute error is considered to be clinically irrelevant because the same treatment will be initiated. Zone B describes the zone where measurement errors are > 0.5 °C but this would not result in a clinical wrong decision. E.g. if T_Eso_ would be 36.2 °C and the TTP would show a temperature of 37.3 °C both temperatures readings would not lead to an intensification of warming therapy or a reduction in temperature delivered by a forced-air warming device. In contrast Zone C indicates errors larger than 0.5 °C that would lead to wrong clinical decisions and may do harm to the patient. e.g. if T_Eso_ would be 34 °C and the TTP would show 37 °C the patient would not receive active warming although this would be indicated.

Data were analyzed using Excel (Microsoft® Excel® 2016, Redmond, WA, USA) and MedCalc® Statistical Software version 19.6.4 (MedCalc Software Ltd, Ostend, Belgium; https://www.medcalc.org; 2021).

## Additional volunteer experiments

To get an insight into how to explain the results, we conducted two volunteer experiments. In these experiments we located branches of the temporal artery with ultrasound and placed one TTP directly above the artery and a second one on the forehead far away from the artery. Then we compared the measured temperatures with T_Eso_ during exposure of the forehead to the environment, during exposure to active warming under a forced-air warming blanket and during active cooling under the forced-air warming blanket.

## Results

### Biometrics and clinical data

**Fig. 1:**
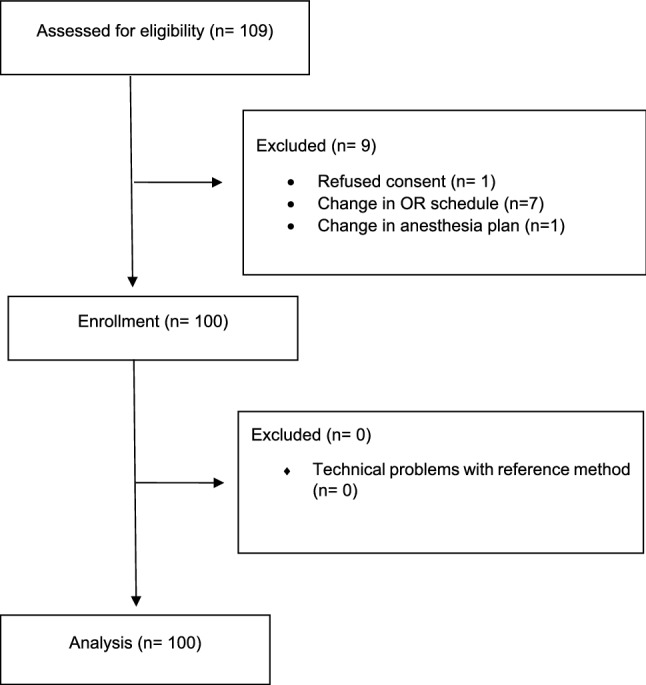
Flow diagram of enrollment

We enrolled 109 subsequent adults undergoing surgery with general anesthesia between January and November 2021. Eight patients were excluded due to organizational changes and one patient refused to participate (see Fig. 1).

3421 data pairs could be analyzed. The participants’ characteristics are presented in Table 1. The measurements of T_Eso_ ranged from 34.0 to 38.3 °C with a mean of 36.4 ± 0.6 °C. T_TTP_ measurements ranged from 33.9 to 37.8 °C with a mean of 36.4 ± 0.5 °C.

**Table 1:** Participant characteristics

Age ± SD	63 ± 16 years
Height ± SD	170 ± 11 cm
Weight ± SD	79 ± 21 kg
BMI ± SD	27 ± 7 kg/m²
Sex	44 Male/56 female
*Type of surgery*	
Vascular surgery	33
Abdominal and urogenital surgery	30
Orthopedic surgery	17
Breast and plastic surgery	13
Head and Neck surgery	7
ASA status (I/II/III/IV)	11/45/41/3
*Anesthesia method*	
Balanced anesthesia	54
TIVA	42
Balanced anesthesia with epidural anesthesia	4
*Warming method*	
Upper body blanket	30
Combined upper body and underbody blanket	12
Underbody blanket	52
Conductive warming	4
Passive Insulation	2

## Bland Altman analysis

**Fig. 2:**
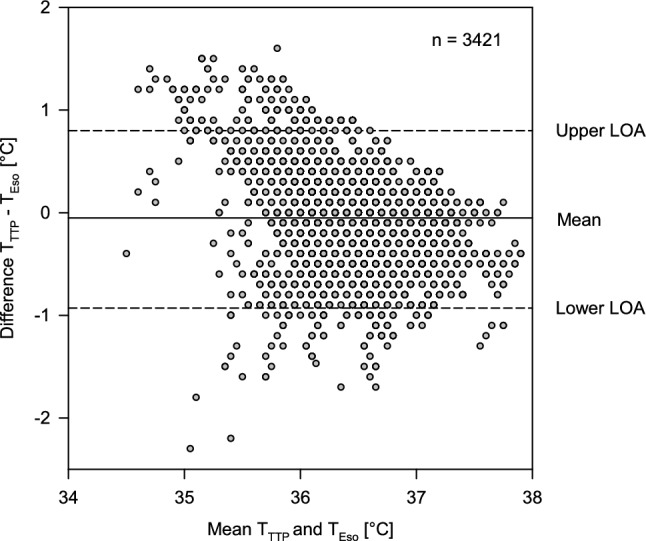
Bland-Altman plot with multiple temperature measurements (100 patients with 3421 measurement pairs) of the TTP sensor (T_TTP_) and an esophageal probe (T_Eso_). Solid line indicates mean bias − 0.04 °C) and dashed lines 95% limits of agreement (LOA). Upper LOA: +0.91 °C, lower LOA: -0.99 °C

Compared to T_Eso_, T_TTP_ measurements resulted in a mean bias of -0.04 °C with 95% limits of agreement within − 0.99 °C [95% CI: − 1.13 to − 0.87 C] to + 0.91 °C [95% CI 0.79 to 1.05 °C] (see Fig. 2). The TTP showed an overestimation of low temperatures and an underestimation of higher temperatures.

### Proportion of differences within ± 0.5 °C and Lin’s concordance correlation coefficient

75% of measured temperature differences where within ± 0.5 °C of T_Eso_. Lin’s concordance correlation coefficient was 0.62 (95% CI 0.59 to 0.63).

#### Sensitivity, specificity, positive and negative predictive values

The calculated sensitivity, specificity, positive and negative predictive values for the detection of hypothermia and hyperthermia are shown in Table 2.

**Table 2:** Sensitivity, specificity, positive and negative predictive values for the detection of hypothermia and hyperthermia

	Sensitivity[%]	Specificity[%]	PPV[%]	NPV[%]
*Detection of hypothermia*				
T_TTP_	31.4	88.9	41.2	83.9
*Detection of hyperthermia*				
T_TTP_	0	100	-	99.5

*T*_*TTP*_ temperature measured with the TTP, *PPV*  positive predictive value, *NPV* Negative predictive value

#### Error grid analysis

**Fig. 3:**
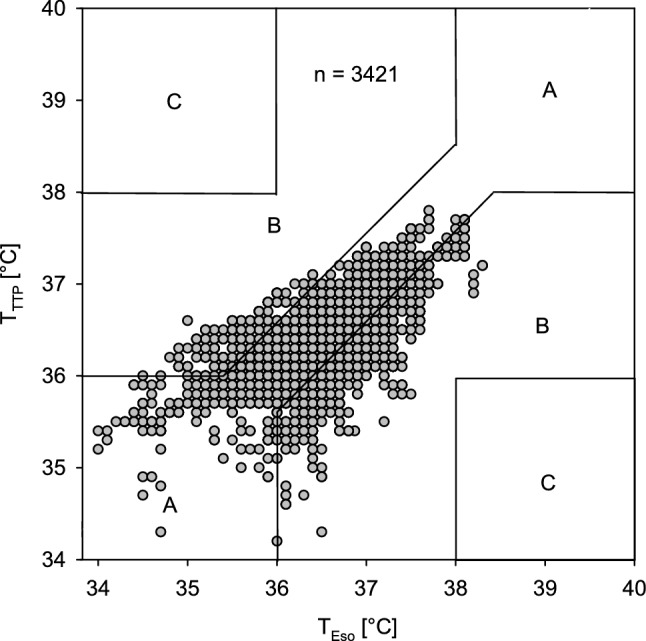
Error grid analysis of the TTP measurements (T_TTP_) versus esophageal temperature (T_Eso_). Zone A is defined as accurate core temperature measurement (< 0.5 °C) or a clinical irrelevant error. Zone B describes the zone where measurement errors are > 0.5 °C but this will not result in a clinical wrong decision whereas Zone C indicates errors that will lead to wrong clinical decisions

Error grid analysis showed that 77.5% of all TTP measurements were clinically not different from T_Eso_ or would lead to the same treatment. In 22.5% measurement errors were > 0.5 °C, but the result would not lead to a clinical wrong decision. None of the measurements would lead to wrong clinical decisions (Fig. 3). The TTP sensor was well tolerated in all patients and no skin lesions was observed.

#### Additional volunteer experiments

**Fig. 4:**
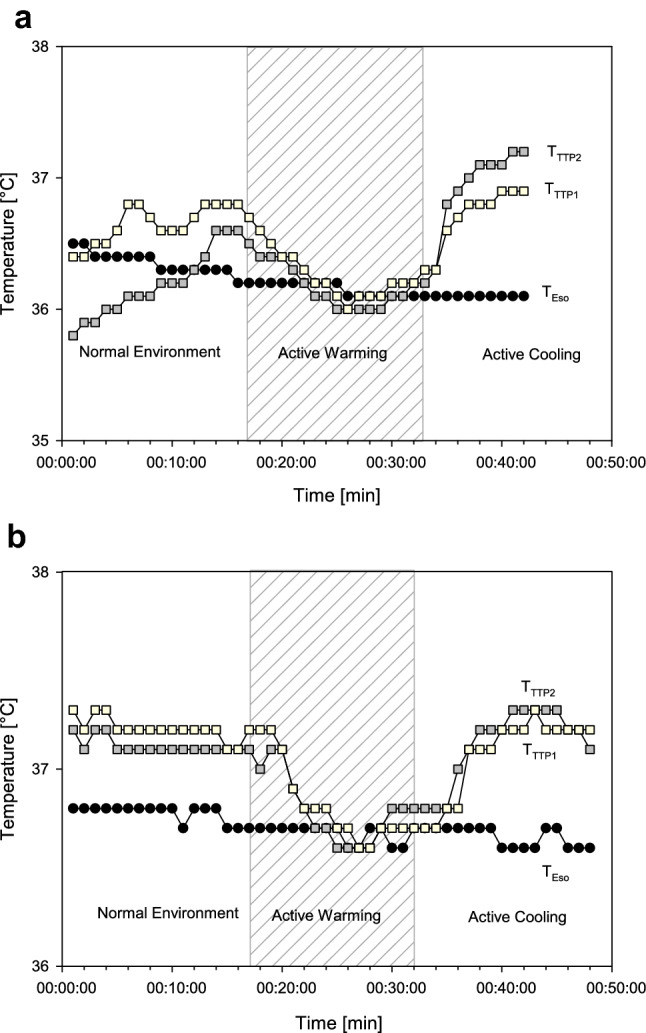
Data of two volunteer experiments (**a** and **b**). TTP measurements above the temporal artery (T_TTP1_) versus TTP measurements far away the temporal artery (T_TTP2_) versus esophageal temperature (T_Eso_)

The results of the additional volunteer experiments are shown in Fig. 4.

## Discussion

The TTP Temperature Monitoring System showed a mean bias of -0.04 °C when compared against the temperature measurement in the distal esophagus in 100 adults undergoing surgery with general anesthesia. This is virtually no difference. However, the TTP Temperature Monitoring System tends to overestimate low core temperatures and tends to underestimate high core temperatures. This can be seen in the Bland-Altman Plot (Fig. 2) but also in the error grid analysis (Fig. 3). In contrast to the minimal bias the limits of agreement were − 0.99 °C [95% CI − to − 0.87 °C] to + 0.91 °C [95% CI 0.79 to 1.05 °C]. As described above, we performed two volunteer experiments to get some insight why the limits of agreement were relatively large. With all caution, based on these two observations, it seems that the location of the TTP thermometer matters for the accuracy of the TTP but even more the exposure of the thermometer to warm or cool air (Fig. 5).

The limits of agreement are higher than the goal that was proposed in an overview article [25] and used in several studies about the accuracy of clinical thermometers [18, 26–28]. In our opinion this objective is very high and most of the studies that have investigated new non-invasive thermometers [18, 26–28] failed to meet this criterion. If we would have used this definition of accuracy, we would also have failed to meet this criterion. Still most of the studies came to the conclusion that these new non–invasive thermometers were accurate enough for clinical practice [18, 26–28] and therefore the NICE guideline recommends the use [13].

When we compare our results to the literature there is only one publication about the TTP to date [21]. In this study with 34 adults and 16 children the authors compared the new non-invasive thermometer with T_Eso_ in 25 patients and with nasopharyngeal temperature in 25 patients. The authors also found no bias and found limits of agreement of − 0.58 to 0.53 °C when comparing the TTP to T_Eso_. Theses limits of agreement are smaller than the limits of agreement that we have found in a much larger cohort of patients. As a consequence, the number of measurements within ± 0.5 °C of the reference method was higher than in our study (92% versus 75%).

To put these results into context it makes sense to compare our results with the evaluation results of other non-invasive thermometers based on heat flux technology like the Tcore™ and the SpotOn™ in adults that compared these methods against T_Eso_. The Tcore™ is a thermometer based on heat flux technology that is applied to the forehead of the patient and not over the temporal artery. In contrast the SpotOn™ is a zero-heat flux thermometer that uses a servo-controlled heater in addition to the heat flux transducer.

The Tcore™ showed in two studies with adult patients also a negligible bias of − 0.01 or 0.08 °C with limits of agreement in the range of − 0.66 °C to 0.59 °C when compared to T_Eso_ [26, 29]. In another recent study in adults after cardiac arrest also a negligible bias of − 0.02 was found. However, the limits of agreement were much larger (− 1.02 to 1.07 °C) [30].

The SpotOn™ was compared in 7 studies against T_Eso_ in adults. The bias was in the range of 0.005 to 0.2 °C and the limits of agreements were in the rage of − 0.55 to 0.73 °C [31–37] when temperature changes were not extremely fast like during application of hyperthermic intraperitoneal chemotherapy.

Therefore, the TTP did not achieve the accuracy of the active SpotOn™ thermometer in our study but was in the range of the results that were found for the passive heat flux thermometer.

Another possibility is to compare the results of the TTP to conventional temporal artery thermometers although these devices use a different technology and allow only spot checks of core temperature and not continuous measurements. To date there are not so many studies available that have compared temporal artery thermometers with T_Eso_. In a small study by Calonder et al. with only 46 measurement points in 22 patients the bias between a temporal artery thermometer and an esophageal temperature probe was 0.07 °C with limits of agreement between − 0.319 and 0.467 °C [38]. In contrast Paik et al. found a bias in the range of − 0.42 to − 0.67 °C and limits of agreement up to 1.24 to − 2.55 °C [39] in a study with 54 patients and much more measurement points. Other well controlled studies in the intensive care unit that have compared temporal artery thermometers versus pulmonary artery catheter found a small bias of − 0.02 °C and limits of agreement of roughly ± 0.9 °C [40] or even a bias of 1.3 °C and limits of agreement of ± 1.2 °C [41]. Therefore, it seems that the TTP is superior to conventional temporal artery temperature measurement.

## Proportion of differences within the range of ± 0.5 °C of the reference method

Another possibility is to look at the accuracy of thermometers is to look at the proportion of differences within the range of ± 0.5 °C of the reference method. In this study 75% of all measurement values of the Temple Touch Pro™ were within the range of ± 0.5 °C of T_Eso_. That is a lower value than reported by Evron et al. [21] who found that 94% of all values were in the range of ± 0.5 °C of their reference methods. Still it seems to be acceptable.

## Diagnostic ability to detect hypothermia or hyperthermia

Sensitivity, specificity, positive predictive or negative predictive values for the detection of hypothermia or hyperthermia were low. This makes correct diagnosis of hypothermia or hyperthermia unreliable. It could be argued that the detection of hypothermia should trigger active warming methods and that the detection of hypothermia is not very reliable. On the other hand, prevention of perioperative hypothermia must start before induction of anesthesia [13] so therefore the precise determination of the hypothermia threshold is not of extreme importance for the management of the patient. However, the precise determination of the hypothermia threshold is important for quality control.

## Error grid analysis

Another interesting way of interpreting the results is the error grid analysis [19]. In this analysis 0% of the measured values would have led to a wrong clinical decision, which also seems to be acceptable.

### Limitations of the study

When evaluating new measurement methods, using the correct reference method is crucial. The best reference method in adults is blood temperature in the pulmonary or iliac artery [17, 19]. However, blood temperature is rarely measured during most surgical procedures. In addition, blood temperature is affected by cold infusions. T_Eso_ is also a so-called gold standard of core temperature measurement [17]. If it is placed correctly it lies between the left atrium and the aorta descendens and is therefore far away from the potentially cooling airway [42]. Although correct placement was attempted by calculating the adequate depth of insertion by the formula of Mekjavic [23] it is still possible that the soft esophageal temperature probe may have been bended in the esophagus and therefore did not have the correct position. It also may have been influenced by upper abdominal surgery [43].

Another potential source of measurement error could be that the TTP was not placed correctly direct above the temporal artery. The recommended placement aims to place the sensor above the arteria zygomaticoorbicularis or the ramus frontalis of the temporal artery that can be tortuous especially in elderly patients. Thus it is possible to miss the right location and thereby get wrong measurements. If this would be true, a modification of the TTP sensor unit could make sense. If the sensor would be larger and would have an array of multiple temperature probes instead of three temperature probes the probability of a placement direct above an arteria would be higher.

Another possible confounder could be perioperative insulation of the head or even the application of warm air to the head thus influencing the TTP.

We also cannot make statements about the accuracy in the presence of severe hypothermia, as we only included elective surgical patients and tried to maintain perioperative normothermia in all patients. Therefore, studies with the TTP in patients with larger intraoperative temperature changes would be worthwhile.

## Conclusions

Non-invasive core temperatures obtained with the TTP in adults showed a mean bias of − 0.04 °C with 95% limits of agreement within − 0.99 °C to + 0.91 °C compared to an esophageal temperature probe. Nevertheless, because measurements with the TTP will not lead to wrong clinical decisions, we consider the TTP as a reasonable tool for perioperative temperature monitoring. It is not accurate enough to be used as a reference method in scientific studies, but may be a useful tool especially for conscious patients undergoing neuraxial anesthesia or regional anesthesia with sedation. Further improvements of the device are desirable and may lead to a higher accuracy.

## Data Availability

Department of Anesthesiology, University Medical Centre Göttingen, Germany.

## References

[1] Coburn M, Rossaint R, Bause H, Biscoping J, Fries M, Henzler D, Iber T, Karst J, Meybohm P, Mierke B, Pabst F, Schälte G, Schiff JH, Stevanovoc A, Winterhalter M (2016). Qualitätsindikatoren Anästhesiologie 2015. Anästh Intensivmed.

[2] Sun Z, Honar H, Sessler DI, Dalton JE, Yang D, Panjasawatwong K, Deroee AF, Salmasi V, Saager L, Kurz A (2015). Intraoperative core temperature patterns, transfusion requirement, and hospital duration in patients warmed with forced air. Anesthesiology.

[3] Karalapillai D, Story D, Hart GK, Bailey M, Pilcher D, Schneider A, Kaufman M, Cooper DJ, Bellomo R (2013). Postoperative hypothermia and patient outcomes after major elective non-cardiac surgery. Anaesthesia.

[4] Schmied H, Kurz A, Sessler DI, Kozek S, Reiter A (1996). Mild hypothermia increases blood loss and transfusion requirements during total hip arthroplasty. Lancet.

[5] Frisch NB, Pepper AM, Rooney E, Silverton C (2017). Intraoperative Hypothermia in Total Hip and Knee Arthroplasty. Orthopedics.

[6] Yi J, Liang H, Song R, Xia H, Huang Y (2018). Maintaining intraoperative normothermia reduces blood loss in patients undergoing major operations: a pilot randomized controlled clinical trial. BMC Anesthesiol.

[7] Bock M, Müller J, Bach A, Böhrer H, Martin E, Motsch J (1998). Effects of preinduction and intraoperative warming during major laparotomy. Br J Anaesth.

[8] Hofer CK, Worn M, Tavakoli R, Sander L, Maloigne M, Klaghofer R, Zollinger A (2005). Influence of body core temperature on blood loss and transfusion requirements during off-pump coronary artery bypass grafting: a comparison of 3 warming systems. J Thorac Cardiovasc Surg.

[9] Kurz A, Sessler DI, Lenhardt R (1996). Perioperative normothermia to reduce the incidence of surgical-wound infection and shorten hospitalization. Study of Wound Infection and Temperature Group. N Engl J Med.

[10] Melling AC, Ali B, Scott EM, Leaper DJ (2001). Effects of preoperative warming on the incidence of wound infection after clean surgery: a randomised controlled trial. Lancet.

[11] Moslemi-Kebria M, El-Nashar SA, Aletti GD, Cliby WA (2012). Intraoperative hypothermia during cytoreductive surgery for ovarian cancer and perioperative morbidity. Obstet Gynecol.

[12] Wang H, Pei H, Chen M, Wang H (2018). Incidence and predictors of surgical site infection after ORIF in calcaneus fractures, a retrospective cohort study. J Orthop Surg Res.

[13] NICE: Addendum to Clinical Guideline 65, Inadvertant Perioperative Hypothermia. (2016). Accessed. 31825571

[14] Torossian A, Bräuer A, Höcker J, Bein B, Wulf H, Horn EP (2015). Preventing inadvertent perioperative hypothermia. Dtsch Arztebl Int.

[15] Link T (2020). Guidelines in Practice: Hypothermia Prevention. AORN J.

[16] O’Grady NP, Barie PS, Bartlett JG, Bleck T, Carroll K, Kalil AC, Linden P, Maki DG, Nierman D, Pasculle W, Masur H, American College of Critical Care M (2008). America IDSo. Guidelines for evaluation of new fever in critically ill adult patients: 2008 update from the American College of Critical Care Medicine and the Infectious Diseases Society of America. Crit Care Med.

[17] Wartzek T, Mühlsteff J, Imhoff M (2011). Temperature measurement. Biomed Tech (Berl).

[18] Eshraghi Y, Nasr V, Parra-Sanchez I, Van DA, Botham M, Santoscoy T, Sessler DI (2014). An evaluation of a zero-heat-flux cutaneous thermometer in cardiac surgical patients. Anesth Analg.

[19] Bräuer A, Fazliu A, Perl T, Heise D, Meissner K, Brandes IF (2020). Accuracy of zero-heat-flux thermometry and bladder temperature measurement in critically ill patients. Sci Rep.

[20] Cobb B, Cho Y, Hilton G, Ting V, Carvalho B (2016). Active Warming Utilizing Combined IV Fluid and Forced-Air Warming Decreases Hypothermia and Improves Maternal Comfort During Cesarean Delivery: A Randomized Control Trial. Anesth Analg.

[21] Evron S, Weissman A, Toivis V, Shahaf DB, You J, Sessler DI, Ezri T (2017). Evaluation of the Temple Touch Pro, a Novel Noninvasive Core-Temperature Monitoring System. Anesth Analg.

[22] von Elm E, Altman DG, Egger M, Pocock SJ, Gotzsche PC, Vandenbroucke JP (2007). The Strengthening the Reporting of Observational Studies in Epidemiology (STROBE) statement: guidelines for reporting observational studies. Lancet.

[23] Mekjavic IB, Rempel ME (1990). Determination of esophageal probe insertion length based on standing and sitting height. J Appl Physiol (1985).

[24] Bland JM, Altman DG (2007). Agreement between methods of measurement with multiple observations per individual. J Biopharm Stat.

[25] Sessler DI (2008). Temperature monitoring and perioperative thermoregulation. Anesthesiology.

[26] Kimberger O, Thell R, Schuh M, Koch J, Sessler DI, Kurz A (2009). Accuracy and precision of a novel non-invasive core thermometer. Br J Anaesth.

[27] Kimberger O, Saager L, Egan C, Sanchez IP, Dizili S, Koch J, Kurz A (2013). The accuracy of a disposable noninvasive core thermometer. Can J Anaesth.

[28] Soehle M, Dehne H, Hoeft A, Zenker S (2020). Accuracy of the non-invasive Tcore temperature monitoring system to measure body core temperature in abdominal surgery. J Clin Monit Comput.

[29] Kimberger O, Saager L, Egan C, Sanchez IP, Dizili S, Koch J, Kurz A (2013). The accuracy of a disposable noninvasive core thermometer. Can J Anaesth.

[30] Janke D, Kagelmann N, Storm C, Maggioni MA, Kienast C, Gunga HC, Opatz O (2021). Measuring Core Body Temperature Using a Non-invasive, Disposable Double-Sensor During Targeted Temperature Management in Post-cardiac Arrest Patients. Front Med.

[31] Jack JM, Ellicott H, Jones CI, Bremner SA, Densham I, Harper CM (2019). Determining the accuracy of zero-flux and ingestible thermometers in the peri-operative setting. J Clin Monit Comput.

[32] Kollmann Camaiora A, Brogly N, Alsina E, de Celis I, Huercio I, Gilsanz F (2019). Validation of the Zero-Heat-Flux thermometer (SpotOn ®) in major gynecological surgery to monitor intraoperative core temperature: a comparative study with esophageal core temperature. Minerva Anestesiol.

[33] Morettini E, Turchini F, Tofani L, Villa G, Ricci Z, Romagnoli S (2020). Intraoperative core temperature monitoring: accuracy and precision of zero-heat flux heated controlled servo sensor compared with esophageal temperature during major surgery; the ESOSPOT study. J Clin Monit Comput.

[34] Tachibana S, Chida Y, Yamakage M (2019). Using the Bair Hugger temperature monitoring system in neck and chest regions: a pilot study. JA Clin Rep.

[35] Boisson M, Alaux A, Kerforne T, Mimoz O, Debaene B, Dahyot-Fizelier C, Frasca D (2018). Intra-operative cutaneous temperature monitoring with zero-heat-flux technique (3 M SpotOn) in comparison with oesophageal and arterial temperature: A prospective observational study. Eur J Anaesthesiol.

[36] Dahyot-Fizelier C, Lamarche S, Kerforne T, Benard T, Giraud B, Bellier R, Carise E, Frasca D, Mimoz O (2017). Accuracy of Zero-Heat-Flux Cutaneous Temperature in Intensive Care Adults. Crit Care Med.

[37] Mäkinen MT, Pesonen A, Jousela I, Paivarinta J, Poikajarvi S, Alback A, Salminen US, Pesonen E (2016). Novel Zero-Heat-Flux Deep Body Temperature Measurement in Lower Extremity Vascular and Cardiac Surgery. J Cardiothorac Vasc Anesth.

[38] Calonder EM, Sendelbach S, Hodges JS, Gustafson C, Machemer C, Johnson D, Reiland L (2010). Temperature measurement in patients undergoing colorectal surgery and gynecology surgery: a comparison of esophageal core, temporal artery, and oral methods. J Perianesth Nurs.

[39] Paik GJ, Henker H, Sereika S, Alexander S, Piotrowski KA, Appel N, Meng L, Bircher N, Henker R (2019). Accuracy of Temporal Artery Thermometry as an Indicator of Core Body Temperature in Patients Receiving General Anesthesia. J Perianesth Nurs.

[40] Lawson L, Bridges EJ, Ballou I, Eraker R, Greco S, Shively J, Sochulak V (2007). Accuracy and precision of noninvasive temperature measurement in adult intensive care patients. Am J Crit Care.

[41] Suleman MI, Doufas AG, Akca O, Ducharme M, Sessler DI (2002). Insufficiency in a new temporal-artery thermometer for adult and pediatric patients. Anesth Analg.

[42] Whitby JD, Dunkin LJ (1969). Temperature differences in the oesophagus. The effects of intubation and ventilation. Br J Anaesth.

[43] Egan BJ, Clark C (2009). A spurious increase of core temperature during laparoscopy. Anesth Analg.

